# A Data‐Driven Step Forward in Risk Stratification for Extreme Hyponatremia in the Emergency Department

**DOI:** 10.1002/kjm2.70309

**Published:** 2026-10-30

**Authors:** Hugo You‐Hsien Lin, Zong‐Ping Weng, Yi‐Wen Chiu

**Affiliations:** ^1^ Division of Nephrology, Department of Internal Medicine Kaohsiung Medical University Hospital Kaohsiung Taiwan; ^2^ Department of Medicine, College of Medicine Kaohsiung Medical University Kaohsiung Taiwan; ^3^ General Division Kaohsiung Medical University Hospital Kaohsiung Taiwan

Hyponatremia is the most common electrolyte disorder encountered in the emergency department (ED), yet the field has long relied on consensus‐based severity bands—mild, moderate, profound, and more recently “severe” (< 120 mmol/L)—that were built for clinical communication rather than derived from the shape of real‐world sodium distributions [1, 2]. In this issue, Golcuk et al. [3] use kernel density estimation (KDE) to let the data itself define the boundary of extreme hyponatremia, identifying a local minimum at 110 mmol/L that separates a rare (0.027%) but strikingly high‐risk phenotype—distribution‐derived extreme hyponatremia (DDEH)—from the remainder of the hyponatremic spectrum. In the propensity‐matched cohort, 28‐day mortality reached 36.1% in DDEH versus 18.0% in non‐extreme hyponatremia and 6.6% in normonatremia, and the association persisted after adjustment for comorbidity and neurological status (Cox HR: 5.46, 95% CI: 1.74–17.17). This is a methodologically innovative and clinically significant contribution that pushes the field beyond one‐size‐fits‐all severity bands, and it prompts a close look at exactly what a data‐driven threshold adds to our understanding of risk at the extreme end of the sodium spectrum.

This distribution‐based approach marks a meaningful methodological advance. Consensus cutoffs impose discrete categories on what is a continuous, and almost certainly nonlinear, risk gradient [4]. By letting an antimode in the density function mark the boundary, the authors avoid the circularity of selecting a cutoff and then testing whether that same cutoff is associated with the outcome it was chosen to predict. Their bandwidth sensitivity analysis showed the lower‐tail antimode moving only modestly (110–114.67 mmol/L) across a range of smoothing parameters—reasonable evidence that the extreme tail is a genuine feature of this cohort's distribution rather than a bandwidth artifact. Notably, 110 mmol/L sits below other cohort‐specific “extreme” cutoffs in the ED literature, such as < 116 and ≤ 113 mmol/L (Table 1) [5], and it remains unclear whether this progressively lower ceiling reflects a true biological gradient or simply stricter case‐mix selection. Nonetheless, KDE‐derived thresholds are, by construction, sample‐dependent estimates of a specific empirical distribution. A single‐center, 5‐year cohort of 227,027 encounters, however large, cannot substitute for replication in independent populations before this boundary can be generalized. The authors are appropriately explicit about this limitation, and we would underscore it here: 110 mmol/L should be read as a hypothesis‐generating, cohort‐specific marker, not a portable clinical cutoff, until it has been independently validated in external, multicenter cohorts.

**TABLE 1:** Reported sodium thresholds for severe/extreme hyponatremia in emergency department cohorts.

Threshold definition	Value (mmol/L)	Basis	Source
Severe hyponatremia (guideline)	< 120	Expert consensus	Spasovski 2024 [2]
Severe hyponatremia (ED case series)	< 116	Cohort‐specific cutoff	Mustajoki 2023 [5]
Most extreme subgroup (ED cerebral edema/ODS study)	≤ 113	Cohort‐specific cutoff	Burst 2026 [6]
DDEH	≤ 110 (sensitivity range 110–114.67)	Kernel density estimation antimode	Golcuk 2026 [3]

Abbreviations: DDEH, distribution‐derived extreme hyponatremia; ED, emergency department; ODS, osmotic demyelination syndrome.

The more consequential question is whether DDEH is a cause of death or a marker of it. A 36.1% 28‐day mortality rate in a group with a median Charlson Comorbidity Index (CCI) of 6 and a Glasgow Coma Scale (GCS) of 14 is compatible with either interpretation. The authors' Cox model—adjusting for CCI and GCS—is a reasonable and transparent attempt to interrogate this, and the fact that the DDEH signal survived adjustment while the non‐extreme hyponatremia signal did not is informative. But with only 61 DDEH patients and a correspondingly wide confidence interval (1.74–17.17), this adjusted estimate should be treated as hypothesis‐generating, not confirmatory—a point the authors make explicitly, and one that a recent methodologic exchange in this literature has pressed hard on. Mannheimer et al.'s propensity‐matched analysis of the Stockholm Sodium Cohort similarly found that excess mortality across the sodium spectrum tracked closely with the underlying diseases precipitating hyponatremia rather than with sodium itself, and the accompanying correspondence from Xia et al. underscores the same point: association with a laboratory value is not evidence that correcting the value corrects the risk [7, 8]. The hematologic findings here—marked microcytosis, relative lymphopenia, and reactive thrombocytosis—are, while nonspecific, more consistent with an underlying chronic‐disease, reduced‐physiological‐reserve phenotype than with an acute osmotic phenomenon, lending indirect support to a marker‐rather‐than‐driver interpretation of DDEH.

This distinction is not merely academic; it changes what a nephrologist or intensivist should do at the bedside. If DDEH is a marker, the task at 110 mmol/L is not simply “correct the sodium” but “recognize this patient as sitting at the extreme tail of multisystem vulnerability” and mobilize commensurate resources—early specialist involvement, closer monitoring, and workup of the underlying driver—rather than anchoring management on the hyponatremia alone. The study is candid about what it cannot tell us: chronicity, volume status, urine studies, and formal Syndrome of Inappropriate Antidiuretic Hormone Secretion (SIADH) or malignancy workup were unavailable, so etiology‐specific correction planning cannot be derived from this dataset—though the low albumin, low total protein, and marked microcytosis in DDEH hint at chronic, malnutrition‐ or liver‐related contributors that guideline‐recommended correction limits (~8 mEq/L per 24 h, tighter in malnourished or hepatically impaired patients) are designed to guard against [2]. That gap matters practically. A recently published multicenter cohort of 13,988 patients with severe hyponatremia (Na ≤ 120 mEq/L) found faster 24‐h correction (> 12 mEq/L) associated with lower—not higher—90‐day risk of death or delayed neurologic injury than slower correction, challenging some of the conservatism in older correction‐rate teaching [9]. Meanwhile, a contemporaneous ED cohort reported that osmotic demyelination syndrome (ODS), though rare (1.3% among severely hyponatremic patients), is not negligible when correction outpaces guideline limits [6]. Reconciling these signals is exactly the individualized, monitoring‐intensive judgment current guidelines still delegate to the bedside clinician rather than a single cutoff [2, 4]. A threshold like 110 mmol/L may usefully flag who needs that intensity of attention; it should not be read as a license to correct faster or slower per se.

Where does this leave the 110 mmol/L boundary? As an early‐warning trigger—analogous to a laboratory‐based early‐warning score—it is plausible and worth testing prospectively, alongside dynamic, ICU‐relevant sodium and severity trajectories rather than a single ED‐presentation value [10]. As a biological threshold, 110 mmol/L remains unestablished, and the useful next step is external, multicenter validation of both the threshold's stability and its incremental discriminative value over existing severity categories and comorbidity indices alone, paired with the etiologic and correction‐trajectory data this cohort lacked. Until then, this study's contribution is not simply a new number to memorize, but a well‐constructed demonstration that the extreme tail of hyponatremia deserves recognition as its own phenotype—and a genuinely promising foundation for the external validation that could carry this data‐driven approach into everyday ED and nephrology‐critical care practice.

## Conflicts of Interest

The authors declare no conflicts of interest.

## Data Availability

Data sharing not applicable to this article as no datasets were generated or analyzed during the current study.
